# Exploring Class‐II PI3K Inhibition for the treatment of Alzheimer’s Disease: Virtual Screening for PI3KC2A Inhibitors

**DOI:** 10.1002/alz.087050

**Published:** 2025-01-09

**Authors:** Chetna Jadala, Michael T Robo, Timothy I. Richardson

**Affiliations:** ^1^ Indiana University School of Medicine, Indianapolis, IN USA; ^2^ Indiana Bioscience Research Institute, Indianapolis, IN USA

## Abstract

**Background:**

Focusing on novel AD treatments, the TREAT‐AD centers offer an array of free research tools, shared via the AD Knowledge Portal in a Target Enablement Package (TEP). This abstract showcases the research conducted by the IUSM‐Purdue TREAT‐AD Center, specifically focusing on Targeting class‐II PI3K’s as a potential breakthrough in AD therapy. Endocytosis within the brain encompasses diverse pathways for internalizing extracellular cargoes and receptors into cells. The prominent routes include clathrin‐mediated endocytosis and phagocytosis. Endocytosis plays a crucial role in processing amyloid precursor protein (APP) leading to abnormal production of Aβ peptides. Recycling endosomes are vital for delivering and eventually releasing Aβ into the brain. Recent research emphasizes the pivotal role of PI3K‐C2α, a class II PI3K member, in regulated endocytosis through its clathrin‐binding domain. Its localization spans clathrin‐coated pits, endocytic vesicles, early endosomes, and the trans‐Golgi network, generating phosphatidylinositol 3‐phosphate (PtdIns(3)P) and/or phosphatidylinositol 3,4‐bisphosphates (PtdIns(3,4)P2) in vivo. Targeting clathrin‐mediated endocytosis by inhibiting PI3K‐C2α, a key regulator in clathrin coated vesicle formation, could be a potential therapeutic strategy against Alzheimer’s disease.

**Method:**

We conducted extensive virtual screenings of vast compound libraries to determine potent small molecules inhibiting PI3K‐C2α. Employing shape‐based screening, and clustering techniques, we identified leading compounds for subsequent in vitro kinase assays. Compounds exhibiting nanomolar activity were selected for further investigation. Leveraging these findings, we conducted Structure‐Activity Relationship (SAR) studies, optimizing analogs to enhance binding affinity and cellular pharmacology.

**Result:**

We have identified novel PI3K‐C2α inhibitors and are in the initial stages of optimization. These compounds exhibit promising target engagement, pending further assessment for biochemical activity and cellular pharmacology. In silico assessments suggest their structures are ideal for CNS drug discovery plans.

**Conclusion:**

Inhibiting PI3K‐C2α stands as a promising therapeutic approach for Alzheimer’s disease. We've discovered unique molecular structures that inhibit the enzyme. Our findings suggest potential probe molecules for validating the target and developing lead compounds for clinical investigations.

